# Cytokines, Signaling and Epigenetic Mechanisms: Shaping the Acute Lymphoblastic Leukemia Microenvironment

**DOI:** 10.3390/cells15050467

**Published:** 2026-03-05

**Authors:** Carolina Simioni, Luca Maria Neri

**Affiliations:** 1 Department of Life Sciences and Biotechnology, University of Ferrara, 44121 Ferrara, Italy; 2 Laboratory for Technologies of Advanced Therapies (LTTA)-Electron Microscopy Center, University of Ferrara, 44121 Ferrara, Italy; 3 Department of Translational Medicine, University of Ferrara, 44121 Ferrara, Italy

**Keywords:** acute lymphoblastic leukemia, microenvironment, leukemic niche, signal transduction, epigenetic mechanisms

## Abstract

Acute Lymphoblastic Leukemia (ALL) is a heterogeneous hematological malignancy in which disease progression and response to therapy are influenced by a complex network of molecular alterations, interactions with the bone marrow microenvironment, and epigenetic modulation mechanisms. Crosstalk between oncogenic, inflammatory, and immunoregulatory signaling pathways, together with epigenetic modifications, contributes to the maintenance of leukemic survival and the development of therapeutic resistance. This review analyzes the role of cytokines and chemokines such as IL-6, TNF-α, and CXCL12, which act as biological biomarkers and key mediators of leukemia niche remodeling, and the main signaling pathways involved in ALL, such as Wnt/β-catenin, JAK/STAT, PI3K/AKT/mTOR, Notch, and BCR, highlighting their functional interconnection with the tumor microenvironment. The role of epigenetics in modulating the dialogue between leukemia cells and stromal components is also discussed. Epigenetic programs govern leukemia’s dependence on stromal support, inflammatory and niche-derived signals, as well as the microenvironment signaling pathways. Overall, targeting leukemia-niche interactions is a crucial strategy for improving outcomes in ALL and to identify potential molecular vulnerabilities, also for developing new therapeutic approaches for the treatment of the disease.

## 1. Introduction

Acute lymphoblastic leukemia (ALL) represents one of the four classes of leukemia, along with acute myeloid leukemia (AML), chronic lymphocytic leukemia (CLL) and chronic myeloid leukemia (CML). Approximately 75% of leukemias in children and adolescents are ALL, while AML, CLL, and CML primarily affect adults. In particular, AML represents the second most common form in children, accounting for approximately 15–20% of childhood leukemias [1,2,3]. ALL could be defined as a clonal hematologic malignancy characterized by a significant proliferation and accumulation of lymphoblasts, in the bone marrow, peripheral blood, and other lymphoid tissues [4]. These lymphoblasts derive from either B-cell or T-cell lineages and are incapable of differentiating into functional immune cells, leading to bone marrow disfunction and, ultimately, systemic disease. ALL is the most common form of cancer diagnosed in children, but it can also affect adults, in whom the prognosis tends to be significantly worse [5]. The global incidence of ALL varies by age, sex, ethnicity, and geographic location [6]. In developed countries, the annual incidence is estimated to be between 1 and 1.5 cases per 100,000 individuals, with a distinct peak occurring in children aged 2 to 5 years. While the overall survival rate for pediatric ALL has improved dramatically over the past decades,—reaching over 85% in some cohorts, adult patients often face more aggressive disease biology and poorer outcomes, with 5-year survival rates below 50% in many cases [7,8]. As previously stated, there are two main immunophenotypic categories for ALL: T-cell ALL (T-ALL), that accounts for the 15–25% of cases, and B-cell ALL (B-ALL), which takes up nearly 75–85% of cases. Hyperdiploidy, the ETV6-RUNX1 fusion, and BCR-ABL1 positive are examples of recurring chromosomal abnormalities and gene fusions that further categorize B-ALL and have different prognostic and therapeutic implications [9,10,11]. Similarly, T-ALL includes several molecular subgroups, including Early T-cell Precursor (ETP) ALL, which is associated with a more immature immunophenotype and poorer prognosis [12,13]. Studying the leukemic niche in ALL is essential because disease progression does not depend exclusively on cell-intrinsic genetic alterations but is profoundly influenced by the bone marrow microenvironment (BMM). Interactions between leukemic cells, stroma, cytokines, and metabolic signals influence survival, quiescence, and therapeutic response, favoring the persistence of resistant subpopulations. Therefore, the niche acts as an active regulator of the disease, representing a critical node for understanding the biology of ALL.

### 1.1. The Normal Hematopoietic Niche and Relevance to Leukemia

Under physiological conditions, hematopoietic stem and progenitor cells (HSPCs) reside in a specialized and spatially organized microenvironment (also called “niche”) within the bone marrow, comprising a repertoire of nonhematopoietic and hematopoietic cells, extracellular matrix (ECM), and soluble, secretory products also called soluble factors [1,14]. The niches coordinate quiescence, self-renewal, differentiation, and mobilization of HSPCs by regulating a complex crosstalk via cell–cell adhesion, chemokines, signal transduction pathways and other extracellular cues [15].

In a healthy bone marrow condition, the hematopoietic microenvironment therefore guarantees a cellular homeostasis through balanced signals that regulate quiescence, differentiation, and survival. The transition to a pathological microenvironment is triggered by the clonal expansion of genetically and epigenetically altered leukemia cells capable of subverting normal cell–cell interactions [16]. Leukemic cells release soluble factors, extracellular vesicles, and metabolic signals that re-educate stromal, vascular, and immune cells, inducing protective pro-leukemic niches [17]. This results in chronic inflammation and epigenetic remodeling of the microenvironment, which stabilize protective niches favorable to survival, proliferation and therapeutic resistance of leukemic cells [18]. Therefore, the BMM is co-opted or remodeled by malignant cells to transform the supportive but balanced niche into a permissive, tumor-favoring environment and condition. While this phenomenon has been more extensively studied in myeloid malignancies, increasing data support a similarly crucial influence of the niche in lymphoid leukemias such as ALL [19,20].

The leukemic tumor microenvironment (TME) is therefore not just a passive “container”, but a dynamic ecosystem in which stromal cells, osteoblasts, osteoclasts, endothelial cells and immune cells are “reprogrammed” by leukemia cells [21].

The dynamic integration and cooperation of the cellular elements and extracellular signals ensures haematopoietic homeostasis and regenerative capacity [19,22]. More importantly, understanding the biological crosstalk is crucial for the development of new therapeutic strategies targeting not only leukemia cells, but also their supporting habitat [23,24].

### 1.2. What Is the Rationale for Studying the TME and How ALL Alters Its Function?

Although ALL is primarily driven by oncogenic mutations and chromosomal rearrangements, the first steps of the disease, as well as its progression and therapeutic resistance, cannot be fully understood without considering the structure complexity of the TME and its contribution. The bone marrow niche provides a complex ecosystem of stromal, endothelial, mesenchymal, and immune cells that constantly interact with leukemic blasts [1,25].

**Figure 1:**
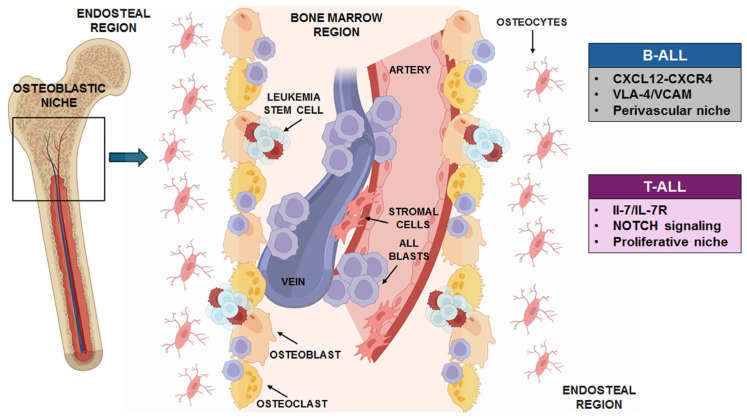
Schematic representation of the bone marrow niche in acute lymphoblastic leukemia (ALL). The boxes summarize the main differences between the ALL niches: while T-ALL cells interact with a more dynamic TME, typified by IL-7/IL-7R and NOTCH-dependent signaling, with higher proliferative activity, B-ALL cells preferentially concentrate in stable perivascular niches. The image was created with Biorender.com.

Moreover, although both B-ALL and T-ALL cells depend on supportive BMM, they establish functionally distinct leukemic niches. Stable perivascular habitats are preferred by B-ALL cells, where adhesion-mediated contacts and C-X-C motif chemokine 12 (CXCL12)-CXCR4 signaling support cell retention, metabolic quiescence, and chemotherapy resistance [25]. This niche is relatively non-inflammatory and mostly makes use of pre-existing vascular and stromal systems. In contrast, T-ALL cells interact with a more dynamic and inflammatory environment, characterized by active niche remodeling, increased proliferative activity, and NOTCH and IL-7/IL-7R-dependent signaling. T-ALL niches are identified by increased cytokine production, enhanced cell motility, and decreased dependence on hypoxic quiescence states [26]. A representative structure of a typical leukemia niche is reported in Figure 1.

Another key feature of the leukemic microenvironment is hypoxia: this condition represents a pathological modifier of the TME, influencing both malignant and stromal compartments to promote disease initiation, progression, therapeutic resistance and immune evasion [27,28]. The hypoxic TME regulates the master transcription factor hypoxia-inducible factor-1α (HIF-1α), that normally induces the activation of glycolytic enzymes (e.g., GLUT1, LDHA, HK2) and suppresses mitochondrial activity, favoring anaerobic environment. In this case, HIF-1α is not hydroxylated and is not degraded by proteasomes, and thus it comes into action. It goes to the nucleus and associates with a similar protein, HIF-1β [29].

Moreover, metabolic crosstalk between leukemic and stromal cells—including exchange of lactate, amino acids, and exosomes carrying microRNAs (miRNAs) further supports leukemic metabolism and survival under therapeutic pressure [30,31].

In addition, signal transduction pathways play a fundamental role in defining and maintaining the leukemic microenvironment, as they regulate the dynamic dialogue between tumor cells and bone marrow stromal cells. Pathways such as JAK/STAT, PI3K/AKT or Notch transmit signals to leukemia cells for survival, proliferation and drug resistance from their surrounding environment [1,24,28,32,33]. These pathways, often hyperactivated or dysregulated, promote the creation of a protective niche that supports leukemia growth and hinders therapeutic eradication [1,32]. Of no less importance, in recent years epigenetic mechanisms have suggested a significant therapeutic potential [34,35]. Epigenetic alterations, particularly DNA methylation and histone modifications, underlie multiple genetic dysfunctions that lead to gene expression dysregulation and promote leukemogenesis [36,37]. The signals derived from the microenvironment, such as cytokines, chemokines, and adhesive interactions, influence the activity of epigenetic enzymes, causing alterations in DNA methylation, histone modifications, and microRNA expression [18,34]. These epigenetic modifications alter the expression of receptors, intracellular mediators and target genes while controlling the sensitivity and activity of important signaling pathways, including Wnt/β-catenin, JAK/STAT, PI3K/AKT, and Notch [38,39]. In this way, epigenetics acts as an amplifier and integrator of microenvironmental signals, contributing to the definition of functional niches that support the survival, quiescence, metabolic adaptation, and therapeutic resistance of leukemic cells. Therefore, understanding how signals are generated, modulated and integrated within the microenvironment urges to seek more and more new therapeutic targets and design strategies that could disrupt these pathological interactions, making leukemia cells more vulnerable to treatment.

## 2. The Role of Cytokines and Soluble Factors in the Characterization of the Leukemic TME: Focus on IL-6, IL-7, CXCL12, TNF-α and TGF-β

### 2.1. Interleukin-6 (IL-6)

Among the most relevant mediators that provide paracrine signals capable of supporting the survival and therapeutic resistance of leukemic blasts, IL-6 acts as a pro-inflammatory cytokine that promotes the proliferation and survival of leukemia cells and significantly modulates the interactions between stromal cells, immune cells and ALL blasts, favoring a permissive environment [40]. High levels of IL-6 in the microenvironment contribute to a chronic inflammatory state that not only promotes tumor growth but also compromises normal haematopoiesis, inhibiting the differentiation of healthy haematopoietic stem cells and allowing leukemia cells to prevail. IL-6 acts primarily by activating JAK/STAT signaling pathways, and its effects on the “malignant niche” are also mediated by the soluble receptor (sIL-6R) through the mechanism of transactivation [41,42]. An observational study of pediatric patients with B-cell ALL showed significantly higher serum levels of IL-6 (along with other pro-inflammatory cytokines) compared to healthy controls, indicating cytokine dysregulation in the context of ALL and suggesting that IL-6 may be a marker of the inflammatory profile of the leukemic TME [43]. IL-6 could therefore have a fundamental relevance in the context of ALL, as it is one of the key molecules through which extracellular vesicles (EVs) mediate disease progression and remodel the BMM. EVs transfer bioactive molecules, such as oncogenic miRNAs (miR-155 and miR-181), into BM Mesenchimal Stem Cells (MSCs) [44].

### 2.2. Interleukin-7 (IL-7)

**Figure 2:**
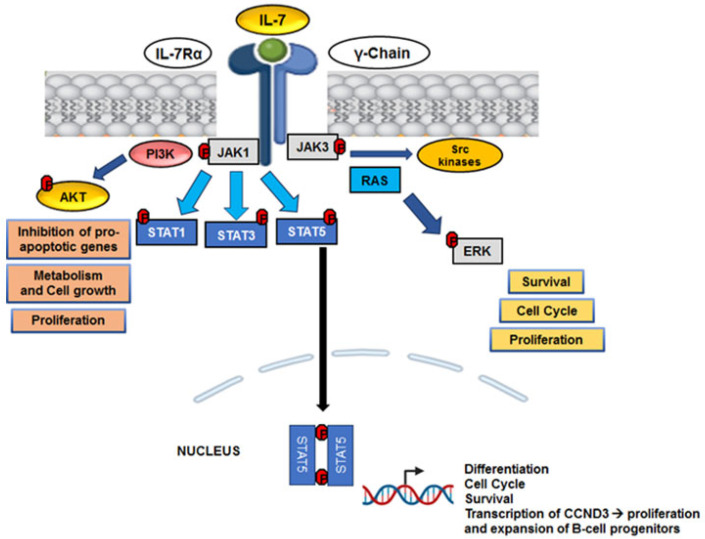
IL-7 signaling in ALL microenvironment. In the leukemic BMM, IL-7 activates its receptor expressed on ALL cells, inducing phosphorylation of JAK1 and JAK3 kinases. Downstream activation of the JAK/STAT (STAT1, STAT3, STAT5), PI3K/AKT, and RAS/ERK pathways, as well as Src kinases, promote transcriptional programs that induce cell survival, cell cycle progression, proliferation, and metabolic reprogramming. In particular, phosphorylated STAT5 translocates to the nucleus and regulates the expression of genes involved in apoptosis inhibition and leukemic growth, contributing to the maintenance of the tumor phenotype supported by the microenvironment. The image was created with Biorender.com.

Interleukin-7 (IL-7) is a crucial cytokine for the survival and metabolism of normal lymphoid precursors, but in the context of ALL it acquires significant pathogenic relevance. In particular, IL-7 is a critical microenvironment-derived cytokine in T-ALL [45]. Numerous studies have shown that IL-7, produced by both stromal cells and microenvironment cells, activates the JAK/STAT, PI3K/AKT and MEK/ERK pathways, promoting proliferation, resistance to apoptosis and protection of lymphoid blasts from therapeutic approaches [46,47,48]. A representative scheme of IL-7 signaling is reported in Figure 2. Adipogenic progenitors, that support leukemic cell growth and their enrichment at relapse, express high expression of IL-7, together with other growth factors and adhesion molecules [49]. As also reported in the study by Kaiser and colleagues, IL-7 induces a strong proliferative burst in early B-cell progenitors (BCPs). In vivo analysis showed that common lymphoid progenitors (CLPs) and pro-B cells from patients with IL-7Rα deficiency had lower expression of the proliferation marker Ki-67 compared to controls, suggesting a proliferative defect in the earliest stages of B lymphopoiesis [50]. At the molecular level, IL-7 stimulates the expression of CCND3 (Cyclin D3) in pro-B3 and pro-B4/pre-BI clusters, suggesting that this gene is central in regulating the proliferation and expansion of the early BCPs pool in response to IL-7R signaling [50]. IL-7 receptor is highly expressed in T-ALL and represents a biomarker for sensitivity to JAK inhibition [45]. Moreover, Buffière and collaborators reported how the ability of T-ALL cells to secrete IL-7 is associated with their epigenetic status. T-ALL cells that produced high levels of IL-7 (>30 pg/10^6^ cells) were significantly less methylated in the IL-7 gene promoter than those that produced low amounts (<30 pg/10^6^ cells) (*p* < 0.01). The methylation status was similar in primary and xenograft samples from the same patients, suggesting that methylation established during leukemogenesis remains stable. The inhibition of IL-7 autocrine secretion delays the development of leukemia by compromising the recruitment and survival of T-ALL cells and reducing the number of Leukemia-initiating cells (LICs) in PDX mouse models [51].

### 2.3. C-X-C Motif Chemokine 12 (CXCL12)

The chemokine CXCL12, also called stromal derived factor 1 (SDF-1) is produced mainly by MSCs, adipogenic progenitors and perivascular cells, both in physiological and pathological conditions [49]. Recent single-cell and transcriptomic studies have demonstrated that this chemokine is not uniformly expressed within the B-ALL bone marrow but rather localizes to discrete stromal niches. In particular, MSC and adipogenic progenitor subsets form CXCL12-rich microdomains that spatially associate with leukemic blasts, promoting their retention, survival, and resistance to therapies [52]. CXCL12 belongs to the “homeostatic” chemokines and is constitutively expressed in specific tissues such as bone marrow, spleen and lung [53,54]. Its production can be significantly regulated in pathological contexts such as hypoxia, via the transcription factor HIF-1 [55,56]. Conversely, signals such as TGF-β can suppress its expression in the primary TME, facilitating the chemotactic migration of CXCR4+ tumor cells to distant sites where CXCL12 is more abundant [57]. The CXCL12/CXCR4 axis orchestrates immunosuppression by chemotactically recruiting suppressive CXCR4+ cells, such as Myeloid-Derived Suppressor Cells (MDSCs), Tumor-Associated Macrophages (TAMs) and regulatory T cells (TREGS), into CXCL12+ niches. This creates a protected “sanctuary” that hinders anti-tumor immune action mediated by cytotoxic T lymphocytes [57]. Therefore, CXCL12 could be interpreted as a key structural element for the localization, retention and protection of leukemia cells, IL-7 acts as a contextual support interleukin, expressed in specific stromal subtypes.

### 2.4. Tumor Necrosis Factor (TNF-α)

As an inflammatory mediator, TNF-α actively participates in the reorganization of the leukemic microenvironment, increasing local inflammation, altering the behavior of stromal cells and enhancing the secretion of additional pro-leukemic factors [58]. TNF-α plays an essential role in creating an ‘inflammatory niche’ that supports the tumor [59]. Indeed, although acute inflammation is a necessary defense mechanism, chronic inflammation can have harmful effects, leading to the development of tumors, HSC depletion, and bone marrow failure, often due to the production of reactive oxygen species (ROS) that damage DNA [60]. TNF-α, together with some other inflammatory mediators, is increased in the bone marrow plasma of B-ALL patients compared to controls and can induce pro-tumor stromal responses, thus contributing to a permissive and immunomodulatory microenvironment [61]. TNF-α promotes angiogenesis and extramedullary infiltration, often associated with leukemia progression. Indeed, it leads to increased expression of pro-angiogenic factors and adhesion molecules (such as VEGF-A and ICAM-1, respectively) and matrix metalloproteinases (MMPs) [62,63]. It has been reported that elevated levels of TNF-α expression are generally associated with adverse clinical parameters and refractory disease [59,63]. Overall, TNF-α is not only an accessory cytokine, but rather, it is a functional indicator of a physiologically active and altered leukemic microenvironment.

### 2.5. Transforming Growth Factor-β (TGF-β)

Transforming Growth Factor beta (TGF-β) directly regulates immune cell differentiation and function, suppressing cytotoxic lymphocyte activity and promoting a microenvironment that is less permissive to tumor eradication [64]. Leukemia-derived extracellular vesicles (EVs) play a crucial role in remodeling the extracellular matrix (ECM) of the bone marrow microenvironment (BMM) [65]. It has also been demonstrated that components of the TGF-β pathway or TGF-β1 secretion can be conveyed or stimulated by EVs in tumor contexts, influencing TGF-β signaling in the microenvironment [65]. At the MSC level, EVs can induce the activation of immunosuppressive programs, with a further increase in the expression of TGF-β1 and target genes of the SMAD/NF-κB pathway [66]. As a result, this condition promotes the survival of ALL cells, with an increase in therapeutic resistance. In pediatric ALL studies on bone marrow biopsies, TGF-β1 expression was found to be increased in megakaryocytes and bone marrow stromal cells, associating with local fibrosis phenotypes and suggesting a possible contribution of the growth factor to structural processes in the ALL microenvironment [67]. Thus, TGF-β, an immunosuppressive molecule carried by EVs and a factor released into the microenvironment, plays a major role in establishing a microenvironment that supports ALL cell dominance and persistence.

## 3. The Signaling Network in the Leukemic TME: Focus on Some Key Signaling Pathways

### 3.1. Wnt/β-Catenin Signaling

The Wnt/β-catenin signaling pathway emerges as one of the main mediators of the dialogue between the BMM and ALL cells, representing a functional axis that integrates niche signals, cell status, and therapeutic adaptation [68]. Since stromal cells, osteoblasts, and components of the bone marrow vascular compartment produce a significant number of Wnt ligands, Wnt signaling in ALL is highly dependent on the microenvironmental context [69]. The niche can directly regulate the identity and destiny of leukemic cells thanks to this paracrine activation, which also maintains a state of functional immaturity and encourages abnormal self-renewal programs. Wnt/β-catenin signaling is unique in ALL because it is linked to leukemic dormancy and quiescence [68]. The persistence of cell subpopulations that are poorly proliferating, found in protective niches, and have a decreased sensitivity to chemotherapeutic therapies has been associated with β-catenin activation [70]. The persistence of transcriptional programs triggered by the microenvironment is further reinforced by the direct and reciprocal interactions of the Wnt/β-catenin pathway with epigenetic processes [71]. After moving into the nucleus, β-catenin modifies the accessibility of Wnt target genes by interacting with chromatin remodeling complexes and epigenetic co-activators. The signal is sensitive to the leukemia cell’s epigenetic state because DNA methylation or histone changes simultaneously control the production of Wnt pathway components and its targets. The niche can convert transient stimuli into stable and adaptable cellular programming because to this interaction [72,73].

### 3.2. The JAK/STAT Signaling

In ALL, the JAK/STAT pathway is constitutively active, promoting oncogenic transformation and increasing cell survival and proliferation [74]. The Ligand–Receptor Complexes that constitute the JAK/STAT network cause JAKs to become activated and phosphorylate particular tyrosine residues on the receptor’s intracellular domain. Signal transducers and activators of transcription proteins (STATs) are phosphorylated by JAKs. These proteins then move to the nucleus and start the transcription of genes, such as the Mcl1 gene, which is essential for B lymphocyte survival. STAT5 is particularly crucial in lymphopoiesis [75,76] and its activation, measurable as pSTAT5, reflects the disease’s dependence on supportive stromal niches and is associated with cell survival and therapeutic resistance [47]. In T-ALL, hyperactivation of the JAK/STAT pathway is frequently due to activating mutations of JAK1, JAK3, and, less frequently, STAT5B, or to autocrine/paracrine signals mediated by cytokines that maintain constitutive phosphorylation of STAT5, contributing to the survival, proliferation, and differentiation blockage of malignant T precursors [77,78]. In B-ALL, particularly in subtypes with Cytokine Receptor-Like Factor 2 (CRLF2) rearrangements (such as Ph-like/BCR-ABL1-like forms), cooperative mutations of JAK2 or activation of the pathway through the formation of the TSLPR-JAK/STAT complex are observed, resulting in a relevant dependence of leukemic cells on STAT5 signaling [79]. Furthermore, even in other B-ALL subtypes, aberrant JAK/STAT activation may be secondary to mutations or amplifications of upstream genes [80]. In both types of ALL, persistent activation of STAT5 promotes the expression of anti-apoptotic genes (BCL-XL, MCL1), cell cycle regulators (CYCLIN D2/D3), and oncogenic metabolic pathways, acting as a functional driver and potential therapeutic vulnerability.

### 3.3. The PI3K/AKT/mTOR Signaling

In addition to the hyperactivation of the JAK/STAT pathway, another well-known signaling pathway of significant importance in the characterization of the ALL TME is PI3K/AKT/mTOR, which is associated with vital processes such as proliferation, metabolism, survival and therapeutic resistance [32,81,82,83]. At the molecular level, the pathway is initiated by phosphatidylinositol 3-kinase (PI3K), which is mainly activated by tyrosine kinase receptors (RTKs) or G protein-coupled receptors (GPCRs). Class I PI3K, which includes the p110α, β, δ and γ isoforms, induces the conversion of PIP2 to PIP3, a secondary messenger that recruits and activates the serine/threonine kinase AKT (also known as protein kinase B or PKB). AKT in turn phosphorylates multiple proteins, including the mTORC1 complex. mTORC1 is essential for the synthesis of proteins, lipids and nucleic acids, while mTORC2 regulates lipid synthesis, the actin cytoskeleton and, in a positive feedback mechanism, can reactivate AKT through the phosphorylation of specific residues [82,84]. The p110 δ and γ subunits of Class I PI3K are significantly expressed in haematopoietic cells and are most involved in leukemogenesis. In particular, in chronic lymphocytic leukemia (CLL), B-cell receptor (BCR)-mediated hyperactivation triggers abnormal activation of PI3K δ, promoting tumor development. In acute myeloid leukemia (AML), the high expression of the p110 δ catalytic subunit and the constant phosphorylation of AKT promote cell proliferation and the survival of neoplastic clones [85,86]. The activation of PI3K/AKT/mTOR is also recurrent in ALL cells [87], and as for other molecules and factors, bone marrow extracellular vesicles (BMM-sEV) transmit signals that activate pro-survival pathways, including PI3K/AKT, in BCR-ABL1^+^ B-ALL, contributing to the aggressive phenotype and poorer prognosis [88]. In T-ALL, the same pathway acts as a hub for integration between microenvironmental signals, particularly the IL-7/IL-7R axis, and intrinsic oncogenic alterations such as PTEN loss or NOTCH1 activation, supporting cell growth, metabolism and chemoresistance [47].

### 3.4. Notch Signaling

Notch signaling has been observed to promote crosstalk between leukemia cells and the bone marrow niche in both B-ALL and T-ALL patients. Indeed, ALL is strongly linked to the dysregulation of Notch signaling. Approximately 60% of patients with T-ALL have a NOTCH1 mutation [89,90]. Furthermore, as for other signaling pathways, Notch signaling may play a role in chemotherapy resistance [91,92]. In Notch-dependent T-ALL models, cancer cells suppress normal hematopoiesis, leading to aberrant B-cell differentiation and myeloid cell growth [93]. Although its involvement in T-ALL is recognized, little is known about its function in B-ALL. In B-cell chronic lymphocytic leukemia (B-CLL) cells, Notch promotes apoptosis and survival resistance. However, even when apoptosis is induced via crosslinking of the B cell receptor (BCR), Notch1 increases cell death but does not alter cell cycle progression in mature cells. Increased susceptibility to apoptosis is therefore not associated with growth or cycle arrest [94,95]. The crosstalk between thymic epithelial cells and different T-ALL cell lines (Jurkat, TALL1, and Loucy), influences Notch signaling and cellular metabolism, regulating the growth and survival of leukemic cells [96]. The results suggest that the thymic epithelial cell (TEC)-conditioned microenvironment can induce apoptosis and cell cycle arrest in more mature Jurkat cells, related to reduced Notch1 activation and changes in the lactate/pyruvate ratio. Furthermore, soluble factors released by Jurkat cells may in turn influence TEC survival and gene expression, highlighting a complex bidirectional interaction crucial to the pathogenesis of leukemia [96]. The take home message is that also this signaling pathway contributes to the characterization of the TME in ALL by integrating stromal signals, particularly the IL-7/IL-7R axis and cooperating with PI3K/AKT/mTOR and metabolic pathways to support the survival, metabolic adaptation and therapeutic resistance of leukemia cells.

### 3.5. BCR Signaling

The B-cell receptor (BCR) signaling pathway is essential for the survival and proliferation of B cells and is abnormally active in some forms of ALL, particularly in pre-B ALL [97].

The LYN and SYK tyrosine kinases are activated when the BCR is activated, resulting in the CD79a/b chains getting phosphorylated. This activates a downstream cascade supporting development and adaptation and activating a number of intracellular pathways, including p38 MAPK, JNK, and RAS/RAF/ERK [98].

By transducing signals downstream of BCR and pre-BCR, Bruton’s Tyrosine Kinase (BTK), a non-receptor cytoplasmic tyrosine kinase, activates effectors such PLCγ2 and promotes the activation of survival and proliferation pathways (AKT, NF-κB, MAPK). BTK controls vital functions like B cell formation, survival, adhesion, migration, and interaction with the microenvironment via various circuits [99].

In B-precursor acute lymphoblastic leukemia (B-ALL), even in the absence of a mature BCR, there is often “BCR-like” or pre-BCR-dependent signaling, in which the PI3K-BTK-AKT axis is functionally active and cooperates with signals from the BMM (CXCL12/CXCR4, integrins). At the transcriptional level, this translates into the activation of factors such as NF-κB, NFAT and FOXO, which regulate genes linked to proliferation and metabolism [20,100,101].

In summary, this pathway functions as a pro-leukemic signaling hub, and for this reason and similarly to the other signaling pathways it represents a potential therapeutic target in selected ALL subgroups.

## 4. The Involvement of Epigenetics Mechanisms in the Characterization of the TME

**Figure 3:**
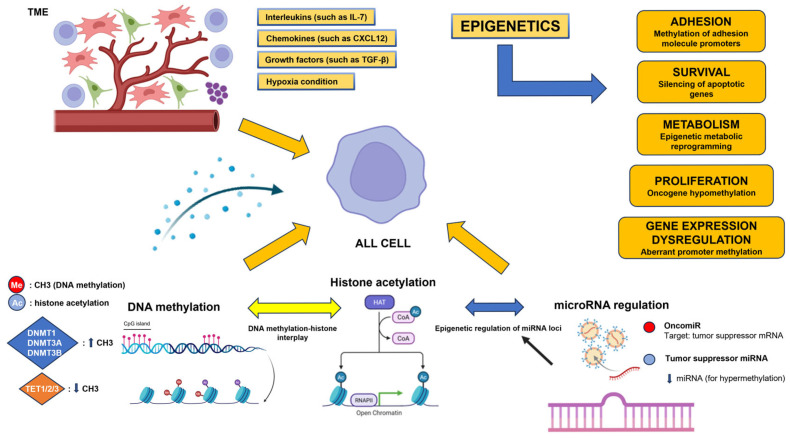
Epigenetic mechanisms contribute to the characterization of the leukemic TME via DNA methylation, histone acetylation, and miRNA-mediated control; signals from the BMM aid in the epigenetic reprogramming of leukemic cells. Interleukins (such as IL-7), chemokines (such as CXCL12), growth factors (such as TGF-β), and hypoxia signals are released by the leukemic BMM, and these substances alter the epigenetic state of ALL cells. These stimuli modify DNA methylation patterns via controlling the activity of TET dioxygenases and DNA methyltransferases (DNMT1, DNMT3A, and DNMT3B). Global hypomethylation encourages oncogenic activation, whereas the hypermethylation of promoter CpG islands silences tumor suppressor genes. Parallel to this, the post-transcriptional dysregulation of gene expression is facilitated by the epigenetic control of microRNAs. These mechanisms facilitate leukemic growth, adhesion, metabolic reprogramming, and survival. This image was created with Biorender.com.

Epigenetic processes are highly relevant in characterizing leukemic TME, as they include key mechanisms through which leukemic cells interact with the surrounding environment, promoting tumor progression, survival and resistance to therapies [18,102] (Figure 3). Unlike genetic alterations, epigenetic modifications are highly plastic and respond dynamically to stimuli from the BMM, such as cytokines, chemokines, hypoxia, and cell–cell interactions [18]. DNA methylation, the first level of epigenetic regulation, is commonly changed at the promoter CpG island level in ALL, and silences differentiation regulators and tumor suppressor genes. Global hypomethylation, on the other hand, contributes to oncogenic activation and genomic instability [38]. A second mechanism is represented by histone modifications, particularly histone acetylation and methylation, which regulate chromatin accessibility and cooperate with DNA methylation in modulating gene expression in response to microenvironmental signals [103]. Finally, microRNA-mediated regulation constitutes a third fundamental epigenetic level, since microRNAs modulate the expression of genes involved in signaling, metabolism, and stress response, and are themselves regulated by DNA methylation and histone modifications [104]. Together, these three epigenetic mechanisms enable the leukemic microenvironment to reinforce survival, quiescence, metabolic adaptation and therapeutic resistance programs, contributing significantly to the definition of functional niches that permit ALL progression (Figure 3).

### 4.1. DNA Methylation

One important epigenetic mechanism in ALL TME is DNA methylation, which translates niche cues into persistent and functionally relevant transcriptional programs. The activity of DNA methyltransferases (DNMTs) and TET dioxygenases is modulated by cytokines, chemokines, hypoxia, and interactions with stromal cells, leading to a dynamic yet long-lasting remodeling of the methylation environment [18]. In order for ALL cells to “memorize” the impact of the microenvironment even in the absence of the initial stimulus, DNMT1, which maintains methylation patterns throughout replication, is essential for combining pro-leukemic transcriptional circumstances. In acute leukemias, higher DNMT1 expression or activity has been linked to quiescence, survival, and resistance to treatment [105]. Additionally, DNMT3A and DNMT3B methyltransferases support the silence of genes involved in differentiation and death by aiding in microenvironment-induced epigenetic rewriting [106]. In the T-lineage setting, where it collaborates with oncogenic and niche signals to drive aberrant self-renewal programs, DNMT3A stands out as a crucial regulator in the development and progression of ALL [107]. Leukemic identity is stabilized by DNMT3B, which also supports the selective hypermethylation of regulatory loci [108]. Active DNA demethylation is mediated by TET dioxygenases (TET1, TET2, and TET3), which add a crucial degree of epigenetic flexibility (Figure 3). The reduced expression or functional inactivation of TET2 and TET1 has been linked to aberrant promoter methylation accumulation and the reinforcement of pathological transcriptional programs in ALL, especially T-ALL, indicating that DNMT–TET imbalance fosters persistence and treatment resistance [109]. TET-dependent loss of control limits the reversibility of epigenetic programs and contributes to establishing states of quiescence, metabolic adaptation, and oncogenic stress tolerance induced by the microenvironment. Together, DNMT1, DNMT3A/3B, and TET1/2/3 constitute an integrated epigenetic axis that allows the leukemic microenvironment to transform transient stimuli into durable cellular identities. This dynamic balance between DNA methylation maintenance and removal explains how the bone marrow niche can favor ALL progression, minimal residual disease (MRD) and therapeutic resistance, making DNA methylation regulation a structural and relevant element in the characterization of the leukemic TME.

### 4.2. Histone Modifications

Histone acetylation represents a crucial epigenetic mechanism for the rapid and reversible regulation of gene expression in response to niche signals [110]. Histone acetylation enables leukemia cells to dynamically respond to BMM stimuli, in contrast to DNA methylation, which helps to stabilize long-term transcriptional programs. The balance between histone acetyltransferases (HATs) and histone deacetylases (HDACs) is altered by these stimuli, with a consequent alteration in the chromatin accessibility, in the regulatory areas of genes related to quiescence, survival, and stress response [111]. Several studies have shown that HDAC activity is dysregulated in ALL, which is linked to the transcriptional silencing of lymphocyte differentiation regulators and tumor suppressor genes [112]. As hypermethylated promoter areas attract methylated DNA-binding proteins, including Methyl-CpG Binding Protein 2 (MeCP2), which in turn encourages the formation of HDAC-containing co-repressor complexes increasing histone deacetylation and chromatin remodeling, this impact is frequently enhanced by interaction with DNA methylation [113]. Acetylation and methylation processes work together to consolidate a repressive transcriptional state, converting transitory microenvironmental cues into long-lasting gene inactivity. At the same time, histone acetylation acts as an integration point for the main active niche-dependent signaling pathways in ALL, including JAK/STAT, PI3K/AKT, and Wnt/β-catenin [114]. Activation of these pathways can induce the recruitment of HATs to specific genomic loci, promoting local hyperacetylation and the expression of pro-leukemic genes, even in the presence of an aberrant methylation context. This dynamic equilibrium allows leukemic cells to maintain remarkable transcriptional plasticity, facilitating adaptation to protective niches, entry into reversible quiescent states, and survival under conditions of therapeutic stress [103].

### 4.3. miRNAs

In addition to methylation and histone acetylation processes, miRNAs are important epigenetic mediators of cellular communication [115]. As is already widely known, miRNAs are non-coding RNAs that regulate gene expression by inducing target mRNA degradation or translational repression [116,117]. miRNA dysregulation is associated with a variety of cancers, including leukemia [34,118,119,120]. Extracellular miRNAs (ECmiRNAs) have been identified as essential intercellular communication mediators that are intrinsic to structural regulation in the TME. In order to satisfy their metabolic and biological requirements, cancer cells utilize miRNAs to modify and form tumor niches, facilitating growth, proliferation, migration, and invasion [118,119,121]. Furthermore, miRNAs are involved in the development and differentiation of ALL [119,122]. Due to the role in integrating signals from cytokines and stromal contacts with intracellular survival and proliferation programs, miR-221 and miR-222 are significant in ALL [123]. These microRNAs are frequently overexpressed in leukemia cells and contribute to the repression of cell cycle and apoptosis regulators, promoting niche adaptation and therapeutic resistance [124]. Research models have demonstrated that ALL cells cultivated with bone marrow stromal cells show cell cycle arrest in the G0 phase and the downregulation of miR-221 and miR-222. Leukemia cells became more sensitive to chemotherapeutic treatments when miR-221 expression is increased, underscoring the function of the microenvironment in controlling these epigenetic variables to affect survival [123]. Different miRNAs also target key genes in signaling pathways that support leukemogenesis in the TME, such as the PI3K/Akt/mTOR pathway [125,126]. In ALL, the up-regulation of the oncomiR miR-21 is associated with repression of the tumor suppressor PTEN and consequent activation of the PI3K/AKT/mTOR signaling network, promoting increased cell survival, proliferation and evasion of apoptosis [127]. By blocking IGF1R and mTOR, miR-99a and miR-100, whose expression is decreased in ALL patients, also function as tumor suppressors [128]. Additionally, a high white blood cell count at diagnosis, T-ALL subclassification, and the presence of important genetic alterations such MLL rearrangement and the BCR-ABL fusion gene are all linked to poor prognostic variables when these miRNAs are not expressed [128]. Inherently in the regulation of the protoncogene Myc and Notch, the miR-17–92 cluster, an important oncogenic component, contains miR-19, which is significantly upregulated in Notch-induced T-ALL. This miR-19 represses target genes such as PTEN, BCL2L11 (Bim), CYLD, HOXA, and NOTCH1 [129,130]. The epigenetic silencing (methylation) of miRNAs may contribute to disease progression [115,131]. For example, miR-203 is silenced by epigenetic processes in BCR-ABL1-positive neoplasms [132]. Cytokine signaling and stromal interactions within the BMM are known to modulate DNMT and HDAC activity, thereby reinforcing pre-existing epigenetic repression programs [18]. In this context, niche-dependent cues may sustain miR-203 silencing and promote the persistence of survival-dependent leukemic clones [16]. Another miRNA, miR-143, is silenced by epigenetic mechanisms in mixed-lineage leukemia (MLL)-AF4-positive ALL, and its restoration can induce apoptosis, offering a potential therapeutic strategy [133,134]. Furthermore, epigenetic alterations are directly implicated in drug resistance, a critical issue in the context of TME [135,136]. The interaction between leukemia blasts and osteoblasts/mesenchymal cells provides chemoprotection (e.g., through adhesion molecules such as VLA-4/VCAM or Wnt/β-catenin activation) and this can be reversed by combining hypomethylating agents (azacitidine) and histone deacetylase inhibitors (panobinostat) with conventional chemotherapy (cytarabine) [137,138]. Dysregulation of the CXCR4/CXCL12 axis, often due to the downregulation of miRNAs (such as miR-101 and miR-139), may also be associated with chemoresistance and metastasis [139]. In addition to miR-326, whose loss facilitates the response to stromal support signals reinforcing dependence on the protective niche [140,141], several miRNAs, including miR-21, miR-155, miR-27a, and miR-125b, are associated with chemoresistance phenotypes in the BMM. These miRNAs regulate the expression of genes involved in the modulation of pathways critical for cell survival and apoptosis, including PI3K/AKT, NF-κB, PTEN and BCL-2, and in the activity of ABC transporters (ABCB1/P-gp, ABCC1/MRP1) [142,143]. A brief summary of the role of these miRNAs is shown in Table 1.

### 4.4. EVs as Mediators for miRNA Transport

EVs, particularly exosomes, represent a key mechanism of the transfer of functionally active microRNAs between leukemic cells and stromal components of the bone marrow in ALL TME [44]. ALL cells release EVs enriched in oncogenic miRNAs, including miR-221/222, miR-155, and miR-146a, which actively remodel the leukemic niche. By suppressing tumor suppressor and apoptosis-regulating genes (including PTEN and CDKN1B), these miRNA-carrying EVs aid in treatment resistance, niche adaptability, and survival [144]. Moreover, studies in hematological malignancies have shown that miRNAs associated with EVs maintain biological activity after transfer and can modulate pathways such as PI3K/AKT, JAK/STAT and NF-κB, highlighting the role of EVs as amplifiers of niche-dependent signals [145]. EVs can also confer drug resistance by transferring resistance pumps (MRP1, P-gp) and miRNAs to chemosensitive cells [146,147]. In ALL, growing evidence indicates that miRNAs transported by EVs play an important role in modulating drug response [44]. By reorganizing transcriptional programs, the EV-mediated transfer of miRNAs from resistant cells to initially chemosensitive cells promotes decreased chemotherapy efficacy and illness duration. Taken together, EV-mediated miRNA transport contributes to cellular plasticity, treatment resistance, and leukemic development, establishing a fundamental level of epigenetic regulation of the tumor microenvironment in ALL.

## 5. Conclusions

This review proposed to summarize a comprehensive overview of cellular and signaling networks that are distinctive of the ALL microenvironment, a dynamic, complex and functionally heterogeneous compartment that contributes significantly to the survival, biological plasticity, and therapeutic resistance of leukemia cells. ALL has a peak incidence in early childhood (≈2–5 years), a developmental window during which the immune system is still maturing. In this phase, immune priming, inflammatory regulation, and the balance between naïve and memory lymphocyte compartments are still being established [148]. Therefore, altered immune regulation during this critical window may facilitate the expansion of pre-leukemic clones or modify bone marrow stromal signaling through cytokine imbalance and immune–stromal crosstalk [30]. Consequently, the expansion of pre-leukemic clones and the early shaping of the leukemic niche may be affected not only by intrinsic genetic and epigenetic mechanisms but also by age-specific immune processes that differ from the more immunologically mature adult setting. In addition, the BMM is highly age dependent [149]. Compared with adults, pediatric BMM displays quantitative and functional differences in stromal/mesenchymal populations and in the expression of key crosstalk molecules, including CXCL12 and Notch ligands [150]. These age-related features may contribute to differences in niche composition and signaling between pediatric and adult ALL. However, a comprehensive analysis of these age-dependent microenvironmental differences is beyond the scope of this review.

Cytokines and soluble factors such as IL-6, IL-7, CXCL12, TNF-α and TGF-β constitute central nodes of communication between leukemic blasts and the stromal compartment, modulating signals that in turn regulate bone marrow retention, metabolic adaptation, immune modulation and the overall creation of a pathological habitat. The CXCL12/CXCR4 axis emerges as a structural element of leukemic bone marrow niches, promoting the anchoring of ALL cells to specific stromal subsets and promoting chemoresistance, dissemination and propagation. IL-7, especially in T-ALL but also in subsets of B-ALL, reinforces niche dependency through the activation of signal transduction pathways, as JAK/STAT5 and PI3K/AKT, while TNF-α and TGF-β mainly contribute to the inflammatory and immunosuppressive microenvironment remodeling. Signal transduction pathways play a key role in characterizing the microenvironment, precisely dictating its transition from a physiological to a pathological state and supporting survival, oxidative metabolism, adaptation to hypoxia and resistance to therapeutic stress.

Understanding epigenetic processes in the microenvironment is also essential because alterations at these levels can be used as diagnostic, prognostic, and predictive biomarkers of treatment response. Methylation, acetylation, and microRNA-mediated regulation, as well as epigenetic plasticity, allow for the modulation of survival, adhesion, metabolism, and stemness pathways in response to microenvironmental pressures [34,151].

In addition, EVs play a crucial role in mediating intercellular communication by transporting leukemic cell microRNAs and other epigenetic modulators to the niche’s stromal and endothelial constituents. Immune evasion, therapeutic resistance, and microenvironmental transformation are all influenced by this vesicle-mediated exchange. All these observations point to EV signaling and epigenetic regulation as key factors influencing microenvironmental heterogeneity in ALL and as potential targets for future therapeutic intervention. From a therapeutic perspective, although molecular classification in ALL has greatly improved because of advancements in genomic technology, not all identified genetic changes result in functional or therapeutically actionable targets. Coordinated multicenter efforts, ethical data governance, and meticulous biological validation are still necessary to guarantee responsible deployment, especially in groups of children that are at risk.

Overall, targeting leukemia–niche interactions is a crucial strategy for improving outcomes in ALL because the interactions between cytokines, signaling pathways and epigenetic mechanisms can characterize a highly adaptive leukemia ecosystem, whose TME not only supports the neoplasm’s progression but also models its biological evolution and therapeutic response.

## Figures and Tables

**Table 1:** miRNAs as epigenetic regulators of leukemic niche adaptation.

miRNA/miRNA Cluster	Main Targets/Pathways	Role in the ALL Microenvironment	Functional Impact	References
miR-221/miR-222	CDKN1B (p27), apoptotic regulators	Integrate cytokine- and stroma-derived signals	- Regulation of cell cycle, survival and niche adaptation; - Context-dependent effects on chemosensitivity	[123,124]
miR-21	PTEN; PI3K/AKT/mTOR	OncomiR involved in microenvironment-driven survival signaling	Enhanced proliferation, survival and apoptosis evasion	[127]
miR-99a/miR-100	IGF1R, mTOR	Tumor suppressor miRNAs downregulated in ALL	Inhibition of growth and metabolic signaling	[128]
miR-17-92 cluster (miR-19)	PTEN, BCL2L11 (Bim), CYLD, HOXA, NOTCH1	Cooperation with Notch signaling in T-ALL	Promotion of survival, proliferation and oncogenic signaling	[129,130]
miR-203	BCR-ABL1–related pathways	Epigenetically silenced in specific ALL subtypes	Contribution to leukemogenesis if silenced	[16,132]
miR-143	Apoptosis-related targets	Epigenetically silenced in MLL-AF4 ALL	Apoptosis if restored	[133,134]
miR-101/miR-139	CXCR4/CXCL12 axis	Regulation of leukemic cell homing and retention in the niche	Chemoresistance and dissemination if dysregulated	[139]
miR-326	Stromal support-related pathways	Dependence on protective niche, when lost	Reinforcement of stromal-mediated survival	[140,141]
miR-21, miR-155, miR-27a, miR-125b	PI3K/AKT, NF-κB, PTEN, BCL-2; ABC transporters	Mediate chemoresistance within the BMM	Enhanced survival, drug efflux and apoptosis resistance	[142,143]

## Data Availability

No new data were created or analyzed in this study.

## Abbreviations

The following abbreviations are used in this manuscript:

ALL	Acute Lymphoblastic Leukemia
AML	Acute Myeloid Leukemia
B-ALL	B-cell acute lymphoblastic leukemia
BCPs	B-cell progenitors
BCR	B-cell receptor
BMM	Bone Marrow Microenvironment
BTK	Bruton’s Tyrosine Kinase
CCND3	Cyclin D3
CDKN1B	Cyclin-Dependent Kinase Inhibitor 1B
CLL	Chronic Lymphocytic Leukemia
CLPs	Common lymphoid progenitors
CML	Chronic Myeloid Leukemia
CRLF2	Cytokine Receptor-Like Factor 2
CXCL12	C-X-C motif chemokine 12
DNMT	DNA methyltransferase
ECM	Extracellular matrix
ECmiRNAs	Extracellular miRNAs
ETP	Early T-cell Precursor
EVs	Extracellular vesicles
GLUT1	Glucose Transporter Type 1
GPCRs	G protein-coupled receptors
HAT	Histone Acetyltransferase
HDAC	Histone Deacetylase
HIF-1α	Hypoxia-inducible factor-1α
HIF-1β	Hypoxia-inducible factor-1β
HSPCs	Hematopoietic Stem and Progenitor Cells
ICAM-1	Intercellular Adhesion Molecule-1
IL-6	Interleukin-6
IL-7	Interleukin-7
LDHA	Lactate Dehydrogenase A
LICs	Leukemia-initiating cells
MCL-1	Myeloid Cell Leukemia-1
MDSCs	Myeloid-Derived Suppressor Cells
MeCP2	Methyl-CpG Binding Protein 2
miRNAs	microRNAs
MMPs	Matrix metalloproteinases
MRD	Minimal Residual Disease
MSC	Mesenchymal Stem Cells
mTOR	Mammalian Target Of Rapamycin
PI3K	Phosphatidylinositol 3-kinase
ROS	Reactive Oxygen Species
RTKs	Tyrosine Kinase Receptors
SDF-1	Stromal Derived Factor-1
STATs	Signal Transducers and Activators of Transcription proteins
T-ALL	T-cell acute lymphoblastic leukemia
TAM	Tumor-Associated Macrophages
TEC	Thymic Epithelial Cell
TET	Ten-Eleven Translocation
TGF-β	Transforming Growth Factor-β
TME	Tumor Microenvironment
TNF-α	Tumor Necrosis Factor- α
TSLPR	Thymic Stromal Lymphopoietin Receptor
VEGF	Vascular Endothelial Growth Factor
